# Regulatory Networks and Complex Interactions between the Insulin and Angiotensin II Signalling Systems: Models and Implications for Hypertension and Diabetes

**DOI:** 10.1371/journal.pone.0083640

**Published:** 2013-12-31

**Authors:** Deniz Cizmeci, Yaman Arkun

**Affiliations:** Department of Chemical and Biological Engineering, Koc University, Istanbul, Turkey; University of North Carolina at Chapel Hill, United States of America

## Abstract

The cross-talk between insulin and angiotensin II signalling pathways plays a significant role in the co-occurrence of diabetes and hypertension. We developed a mathematical model of the system of interactions among the biomolecules that are involved in the cross-talk between the insulin and angiotensin II signalling pathways. We have identified several feedback structures that regulate the dynamic behavior of the individual signalling pathways and their interactions. Different scenarios are simulated and dominant steady-state, dynamic and stability characteristics are revealed. The proposed mechanistic model describes how angiotensin II inhibits the actions of insulin and impairs the insulin-mediated vasodilation. The model also predicts that poor glycaemic control induced by diabetes contributes to hypertension by activating the renin angiotensin aystem.

## Introduction

Diabetes, hypertension and cancer affect a great fraction of people worldwide. Clinical and pharmacological data suggest that these diseases are related [1]–[8]. This study analyzes the structure and the dynamics of the complex cellular networks associated with the cross-talk between diabetes and hypertension.

Biological actions are carried out through complex dynamic interactions of many cellular agents. Insulin stimulates multiple signalling pathways to regulate glucose homeostasis, vascular tone, and cell growth. Deregulation of processes downstream of insulin may result in diseases such as diabetes, hypertension and cancer [9]. AKT (also known as protein kinase B) is an important molecule in the insulin signalling pathway. AKT is activated through a PI3K dependent mechanism and promotes glucose uptake by translocating GLUT-4 to the cell surface [9]–[14]. Activated AKT drives cell proliferation [12] and also enhances vasodilation by stimulating NO production [15], [16]. Insulin resistance can develop through impairments in signalling events involved in activation of AKT. Angiotensin II (Ang II) contributes to the pathogenesis of insulin resistance by inducing inhibition of key signalling elements of insulin AKT pathway [15]. At the same time, diabetes induced hyperglycemia may lead to hypertension by activating Ang II. The renin angiotensin system (RAS) is found to be activated in hypertension and Ang II is an essential vasoconstrictor hormone in RAS [17]. Altogether, these findings suggest that the cross-talk between insulin AKT and Ang II signalling pathways plays a significant role in the co-occurrence of diabetes and hypertension.

This study aims to provide a better understanding of operating principles of processes such as glucose uptake, cell proliferation, and blood pressure control by developing mathematical models of interactions at the system level. System behavior is analyzed within the context of signalling pathways and feedback regulation. We show that complex signalling pathways that govern the cross-talk between hypertension and diabetes are regulated by feedback structures that are organized in hierarchical fashion. Using the dynamic models we develop, we simulate different scenarios to elucidate the functions of these feedback structures. While doing so, dominant steady-state and dynamic characteristics that determine the normal and diseased states are revealed.

## Models

Before we present the mathematical model and the results, it is important to describe the methodology first. Signalling pathway structures are constructed based on established literature knowledge. Known regulatory relations and interactions between biomolecules are integrated to the model systematically. Wherever it is necessary and makes sense, molecular interactions are lumped together to reduce the complexity of the cellular network. Feedback loops are identified as well. Finally, the chemical reaction network of the biomolecules is modelled using mass-action kinetics and conservation laws. The resulting model represents a nonlinear dynamical system, which is in the form of ordinary differential equations. In the following, the physical description of the pathways of interest is given before the analytical model is presented.

Insulin AKT signalling pathway and renin angiotensin systems are standard pathways. It is also well-known that these pathways interact with each other. However, studies reported in the literature do not present the available knowledge in a coherent picture that is amenable to modeling and analysis. We have carefully selected the most relevant cross-talk interactions that are reported in the literature and presented in pathway databases such as KEGG [18]. We have constructed a pathway structure, which is able to reproduce known physiological behaviors. In the sequel, we explain the employed interactions in detail and illustrate the pathway structure upon which the mathematical modeling and subsequent analysis is based.

### Insulin AKT Signalling Pathway

**Figure 1:**
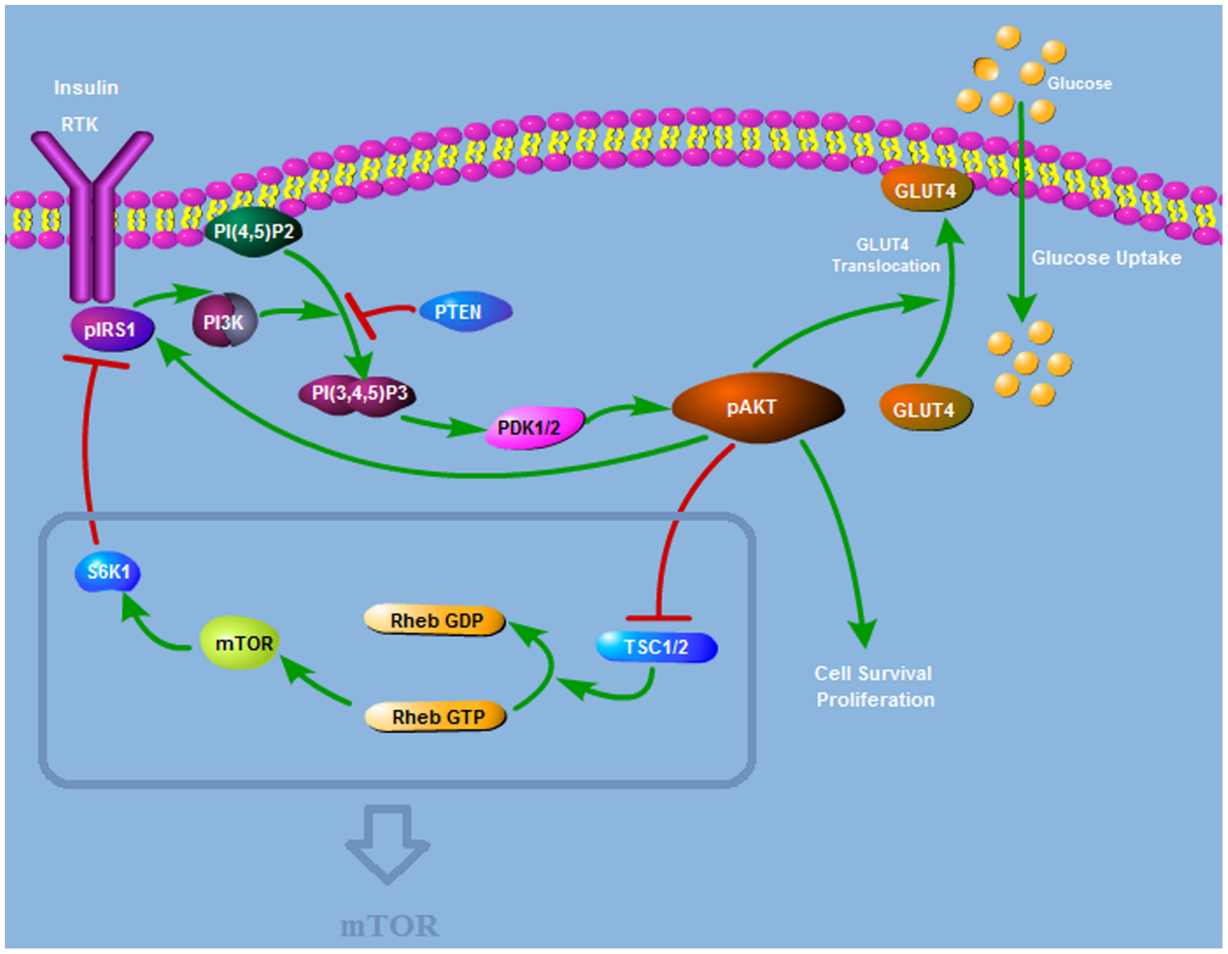
Insulin AKT Signalling Pathway Structure. AKT is activated by PDK1 and PDK2 downstream of insulin. Activated AKT (pAKT) enables the translocation of glucose transporter-4 (GLUT-4). pAKT activates mTOR by phosphorylating the tuberous sclerosis complex (TSC). mTOR activates S6K which phosphorylates and inhibits IRS1.

The pathway is illustrated in Figure 1. In all the pathway diagrams to follow, nodes represent the molecules and links or edges denote the interactions between these nodes. Directed edges with arrows stand for activation and edges with hammer heads represent inhibition. AKT is activated by the events downstream of insulin and its activation is essential for insulin mediated glucose transport. This activation is mediated by PI3K, and mTOR is linked to the pathway to make it sensitive to nutrients.

This pathway can be retrieved from KEGG PATHWAY database. The pathway data has been verified with different sources and simplified for the sake of focusing on the essential paths that give rise to the biological phenomena under study.

Growth factors like insulin, insulin-like growth factor IGF-I, and IGF-II activate insulin/IGF-I receptor tyrosine kinases (RTKs) on the cell surface. Activated RTKs autophosphorylate and create phosphotyrosine binding sites for the insulin receptor substrate (IRS). IRSs are phosphorylated by insulin/IGF-I RTKs on tyrosine residues. Phosphorylated IRSs act as binding sites for proteins containing src homology 2 domains, including the p85 regulatory subunit of class I phosphoinositide 3-kinase (PI3K) [13]. PI3Ks generate PIP3 from PIP2. Subsequently, PIP3 recruits AKT and 3-phosphoinositide-dependent kinase 1 (PDK1) by binding to their PH domain. AKT is activated by phosphorylation on Thr308 and Ser473 via PDK1 and PDK2, respectively [11]. PP2A dephosphorylates AKT on both Thr-308 and Ser473 sites. PHLPPs also dephosphorylate AKT. The lipid protein phosphatase PTEN negatively regulates AKT activation by converting PI3K-generated PIP3 into PIP2, thus blocking AKT phosphorylation on both Thr308 and Ser473 by PDK1 and PDK2, respectively [12].

Plasma glucose homeostasis is maintained during feeding and fasting by regulating the absorption from the intestine, the storage and the release by the liver and the availability for cell uptake via insulin, which is produced in the pancreas, and metabolism by the cells [9]. Activated AKT (pAKT) enables the translocation of glucose transporter-4 (GLUT-4) from cytosol to the plasma membrane, thus glucose is taken into the cell [10], [14]. By stimulating the recruitment of GLUT-4 to the cell surface, pAKT plays a key role in the most significant metabolic action of insulin, which is the glucose uptake.

IRS1 is phosphorylated on serine residues by the activated AKT. This phosphorylation protects IRS1 from the action of protein-tyrosine phosphatases (PTPases) and prevents its dephosphorylation [19]. PTPases dephosphorylate the insulin receptor. PTP1B is found to be upregulated in insulin resistant cells [20]. pAKT negatively regulates PTP1B and prevents dephosphorylation of IRS1 by PTP1B [21]. As a result, AKT positively regulates IRS1 function since IRS1 maintains its tyrosine phosphorylated active conformation.

Nutrient availability is sensed [22], [23] and regulatory signals are transmitted through mTOR (mammalian Target of Rapamycin). pAKT activates mTOR by phosphorylating the tuberous sclerosis complex (TSC), heterodimer of hamartin (TSC1) and tuberin (TSC2). The TSC1/2 complex controls the balance between two forms of a small GTPase called Rheb. Rheb-GDP is the inactive form whereas Rheb-GTP directly activates mTOR. AKT-dependent phosphorylation of TSC2 inactivates the TSC complex and thus the conversion to the inactive Rheb-GDP form is inhibited [24]. Since active Rheb-GTP form is favored, mTOR becomes activated. mTOR activates S6K which phosphorylates and inhibits IRS1 [25]–[27].

### Cross-talk Between Angiotensin II and Insulin Signalling Pathways

Renin is secreted from the kidney and it cleaves angiotensinogen, which is synthesized in the liver, to produce angiotensin I. Then angiotensin converting enzyme (ACE) converts angiotensin I to angiotensin II [28]. Angiotensin II is the active peptide in RAS which plays an important role in controlling blood pressure. It can directly elevate the arterial pressure by rapid vasoconstriction in many areas of the body [17]. Another aspect of the regulation of blood pressure is related to the ability of angiotensin II to affect sodium and extracellular fluid homeostasis by the stimulation of aldosterone production. Aldosterone increases reabsorption of ions and water in the kidney, increasing blood volume and, therefore, increasing blood pressure.

Insulin can mediate vasodilation through AKT activation and the consequential NO production. Vasodilator effects of insulin are mediated by the signalling pathway involving IRS-1/PI-3 kinase/AKT/eNOS that leads to increased NO production by endothelium [16]. AKT leads to phosphorylation of eNOS on serine 1177. eNOS, endothelial nitric oxide synthase, catalyzes the production of nitric oxide from L-arginine.

**Figure 2:**
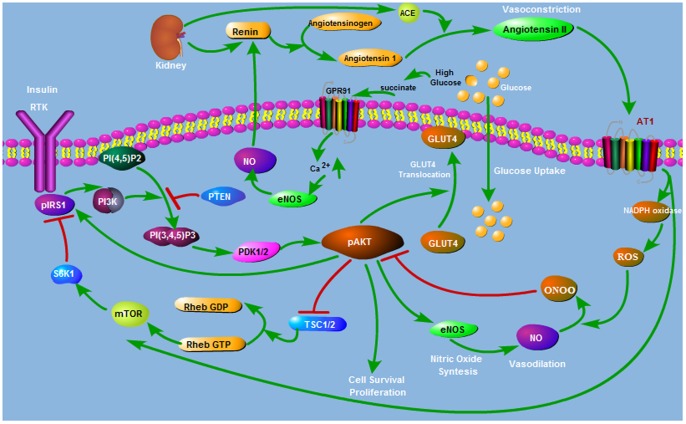
Interaction Between AKT and Ang II Pathways. Renin Angiotensin System (RAS) interacts with insulin signalling through several mechanisms.

Ang II impairs insulin signalling pathway through several mechanisms as shown in Figure 2. Ang II is known to generate ONOO-[29]–[33]. Ang II stimulates production of reactive oxygen species (ROS) such as superoxide O2- through activation of NAD(P)H oxidase [34]–[36]. When both O2- and NO are synthesized, they will react spontaneously to form peroxynitrite (ONOO-) [14]. ONOO- nitrates AKT and prevents its phosphorylation on Ser473 and Thr308 and thus inhibits its catalytic activity [37]. Inhibitory S-Nitrosation of AKT in response to ONOO is shown in [38], [39]. Thus, Ang II would affect insulin-stimulated production of nitric oxide negatively since it leads to inhibition of AKT activation [15]. In addition, Ang II inhibits insulin metabolic signalling and promotes insulin resistance through activation of the (mTOR)/S6 kinase 1 (S6K1) mediated IRS-1 serine phosphorylation [40].

NO regulates renin synthesis and has stimulatory effect on renin secretion and Ang II activation through a cGMP mediated mechanism by inhibiting cAMP degradation [41].

Recently, it is proposed that there exists a direct link between high glucose levels and renin activation mediated by the G-protein-coupled receptor GPR91 and succinate [42]. High glucose levels lead to accumulation of succinate which activates GPR91. Through the activation of GPR91 endothelial cytosolic calcium is increased causing production of prostaglandins and NO. Prostaglandins and NO activate renin release from the juxtaglomerular granular cells (JGG) [41]. Glomerular hyperfiltration and JGG renin activation are observed in diabetes. Since prostaglandins and NO are vasodilator agents that cause relaxation of the afferent arteriole, they can also explain the development of hyperfiltration [42].

### Embedded Regulatory Feedback Structures

Frequently biological networks consist of several feedback loops that are coupled with each other. Complex systems such as biological systems perform their functions through coordinated action of several feedback mechanisms. In [43] it is demonstrated that coupled feedback loops enforce actions which are not possible by single feedback loops. It is shown that coupled positive feedbacks can amplify signals and induce bistability; coupled negative feedbacks maintain homeostasis; coupled positive and negative feedbacks strengthen regulation capacity for better responses regarding time and magnitude. In [43], [44] it is argued that signalling networks consist of coupled feedback loops to enhance robustness of the system. In this paper, nested feedback loops are studied to investigate their role in regulatory mechanisms such as glucose uptake and vasodilation-vasoconstriction balance. Next, we elucidate the feedback loops present in the insulin signalling and angiotensin II pathways.

**Figure 3:**
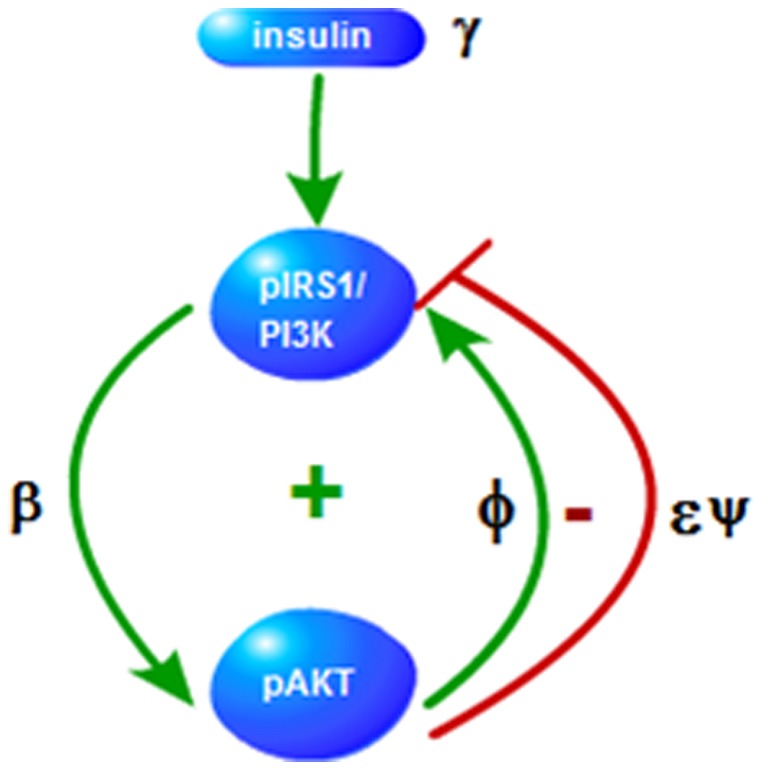
Insulin AKT Signalling Feedback Loops. Signs + and − indicate positive and negative feedback loops, respectively. Parameters (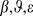) on the edges denote the strengths of the edges. 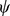 is the nutrient level sensed my mTOR.

Figure 3 shows a simplified version of the insulin AKT signalling pathway after lumping some of the molecular interactions that are explicitly shown in Figure 1. This is similar to the AKT pathway structure given in [45]. The simplified pathway consists of one positive and one negative feedback loop, which are explained next.

#### pIRS1→pAKT→pIRS1 positive feedback loop

pAKT is activated downstream of insulin. In turn, pAKT positively regulates pIRS1 function since it helps IRS1 maintain its tyrosine phosphorylated active conformation [19] and forms a positive feedback loop. Glucose uptake should be sensitive to insulin and this positive feedback regulation ensures the required bistable response [46]. pAKT should switch between high and low values according to the cellular and extracellular conditions since glucose uptake, translocation of GLUT4 to the plasma membrane, is an all or none process [46].

Second property associated with AKT is cell proliferation. Cell proliferation operates also in all or none manner which are controlled by insulin. Insulin resistant systems that cannot switch between on-state and off-state lead to diseases. Systems with persistently low pAKT values are associated with type II diabetes whereas systems with persistently high pAKT values are associated with cancer [45].

#### pIRS1→pAKT→pIRS1 negative feedback loop

pAKT also forms a negative feedback loop by inhibiting pIRS1 through activation of mTOR (see Figure 1). Coupling of the negative feedback with the positive feedback enables the fine adjustment of the response dose and provides a stable regulatory response to nutrient level and helps maintain glucose homeostasis. mTOR is involved in many signalling pathways and especially plays significant role in cell growth, proliferation, survival and protein synthesis. mTOR pathway is found to be activated in cancer and drugs that inhibit mTOR are used for anticancer therapy [47], [48]. As opposed to its cancer promoting effects via other signalling pathways, in this particular system, mTOR acts to reduce cancer. Through inhibition of pIRS1, mTOR decreases the level of pAKT which is one of the agents responsible for cell proliferation. In other words, inhibition of mTOR may reduce cancer through other mechanisms which are not considered in this study, but does drive cancer progression through elevating pAKT levels and driving uncontrolled cell proliferation. This can explain the findings that mTOR inhibition for cancer treatment being less effective than expected [49]. In summary, the relative strengths of the pIRS1 positive and negative feedback loops determine the phenotypes of the system such as normal, diabetes, or cancer.

**Figure 4:**
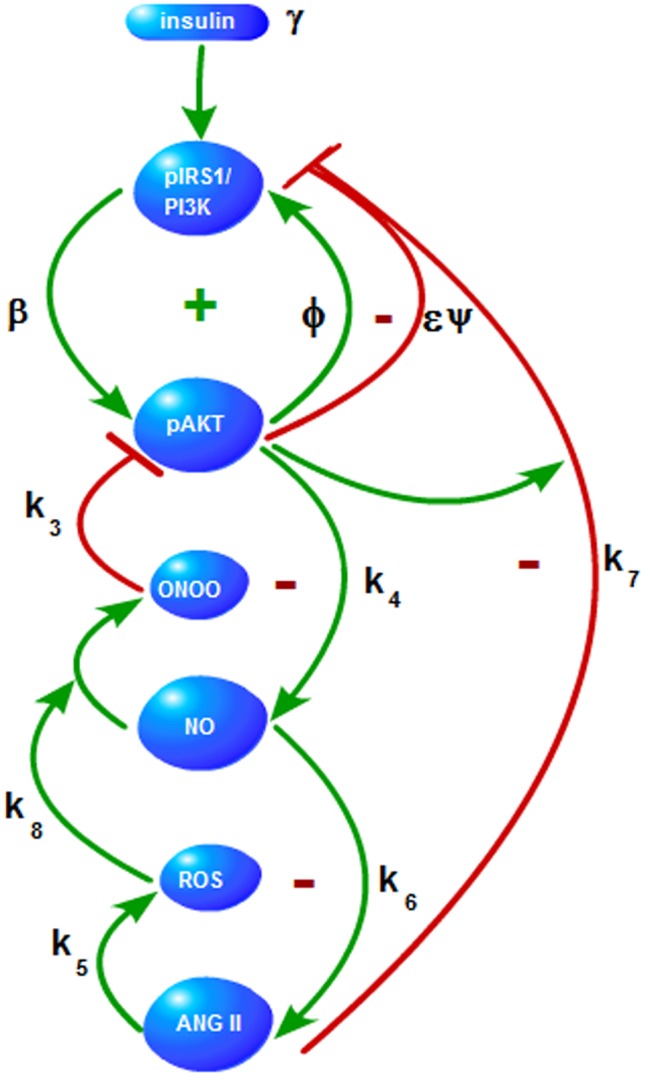
AKT-Angiotensin II Feedback Loops. A hierarchy of feedback loops defines the interactions between the AKT and Ang II signalling pathways.Interaction parameters are labeled on the directed edges between the interacting nodes.

The pathway shown in Figure 2 includes several interactions (i.e. cross-talk) that are present between AKT and angiotensin signalling pathways. The feedback loops underlying these interactions can be organized in a hierarchy as depicted in Figure 4 and listed in Table 1. Signs + and − indicate positive and negative feedback loops, respectively. The first two loops at the top of the hierarchy are the positive and negative feedback loops of the basic AKT system and are already discussed above. Other feedback loops are introduced through the coupling with the angiotensin system and are explained next.

**Table 1:** Feedback Loops.

+	pIRS1→pAKT→pIRS1
_	pIRS1→pAKT→pIRS1
+	pAKT→NO→ONOO→pAKT
_	NO→ANG II→ROS→NO
+	pAKT→NO→ANGII→ROS→ONOO→pAKT
_	pIRS1→pAKT→NO→ANG II →pIRS1

A hierarchy of feedback loops defines the interactions between the AKT and Ang II signalling pathways.

#### pAKT→NO→ONOO→pAKT negative feedback loop

Vasodilator actions of insulin are mediated by nitric oxide (NO). pAKT stimulates NO production through pAKT → eNOS →NO path. NO participates in the production of peroxynitrite *ONOO*, which prevents phosphorylation of AKT and thus inhibits pAKT.

#### NO→ANGII→ROS→NO negative feedback loop

NO is converted to ONOO by the actions of ANG II. Conversion of NO to ONOO is important for preventing the accumulation of NO [50]. Thus this feedback loop helps to balance the NO level. However, when Ang II is overactive, such as in hypertension, NO will decrease through this mechanism. Since NO is a vasodilator agent, decreasing NO levels would further increase the blood pressure.

#### pAKT→NO→ANG II→ROS→ONOO→pAKT negative feedback loop

NO stimulates renin production and thus activates Ang II. Activation of Ang II produces reactive oxygen species ROS and ONOO which inhibits pAKT.

#### pIRS1→pAKT→NO→ANG II →pIRS1 negative feedback loop

Stimulation of NO production downstream of pIRS1 contributes to IRS1 inhibition by activating Ang II. Ang II promotes insulin resistance and inhibits insulin metabolic signalling by activating mTOR and thus promoting S6K1-mediated IRS-1 serine phosphorylation [40].

Figure 4 suggests that the key internal states of the system are pAKT and NO. Proper regulation of pAKT and NO by the positive and negative feedback loops is crucial in maintaining the right balance between glucose uptake and vasodilation-vasoconstriction effects.

### Modeling Background

In [45] Wang developed a mathematical model for the AKT signalling pathway to investigate system-level mechanisms of cell growth and metabolism. The model centers on mTOR which senses the level of nutrients, insulin growth factor which activates the pathway, and pAKT (phosphorylated AKT) that adjusts the growth. Modeling equations are based on the phosphorylation and dephosphorylation cycle (PdPC) of AKT → pAKT:
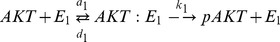


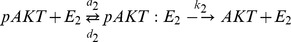
where E_1_ and E_2_ are the enzymes. Assuming Michaelis-Menten kinetics, the following dynamical model is derived [45]:



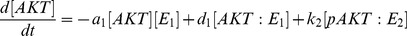
(1)

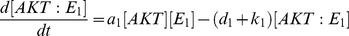
(2)

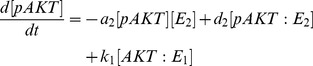
(3)

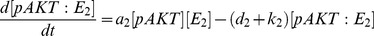
(4)

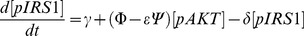
(5)with
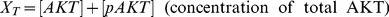
(6)

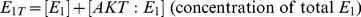
(7)

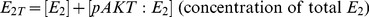
(8)

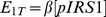
(9)


Variable 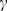 denotes the insulin level and 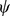 is the nutrient level. The critical parameters 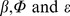 represent the strengths of the feedback loops as shown in Figure 3. The rest of the model parameters are constant physical parameters representing the reaction rates and the decay rate of pIRS1. It was shown that the steady-state solutions were determined by the insulin level, 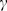, relative strengths of the positive and negative feedback loops defined by 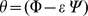, and the Michaelis-Menten kinetic constants, 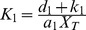 and 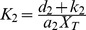.

Wang studied the above model under steady-state conditions by setting equations (1) – (5) equal to zero.

Steady-state response curve between pAKT (output) and insulin (input) was calculated for different model parameter values which led to the partitioning of the parameter space into regions for the normal response and disease phenotypes including cancer and diabetes. It was shown that the normal phenotype corresponds to two steady states between which the system is able to switch. It is further shown that the switch between these steady states is possible when bistability (existence of two stable steady-states) and high sensitivity to the input insulin are present.

**Figure 5:**
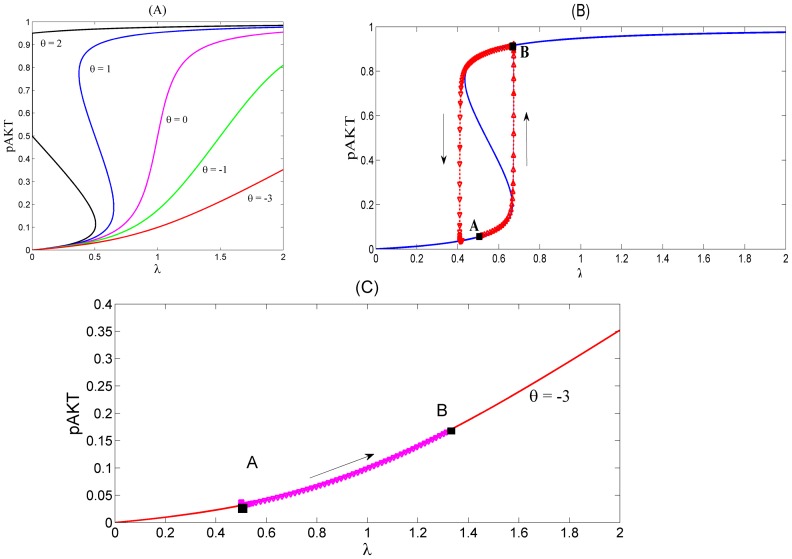
Response Curves of the AKT model. Insulin level is redefined as 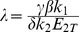 [45]. (**A**) Steady-state response curves of pAKT vs insulin 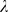 for different feedback strengths 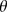. 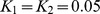. The curves are calculated by setting equations (10) and (11) equal to zero. (**B**) Dynamic response curve in red superimposed on the steady-state curve shows the normal insulin cycle between states A and B. 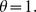 At State A, the cell has low nutrient level and requires glucose uptake. By stimulating insulin, the system switches to State B, where pAKT is activated and glucose is taken into the cell. Withdrawing insulin enables the switch back to low pAKT levels. (**C**) Bistability is lost under excess negative feedback 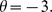 Steady-state curves in (A) are identical to those in [45]. Dynamic responses in (B) and (C) are new and they are generated form the dynamic model equations (10) and (11).

For example Figure 5 (A) shows the steady-state responses of pAKT to insulin for different values of the feedback strength parameter 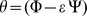. Normal operation of the insulin signalling pathway requires the positive feedback to be greater than the negative feedback i.e. 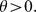 For example, when 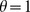, the system is responsive to a small change in the insulin level and is able to switch both ways between high and low pAKT values as shown in Figure 5 (B). This toggle switch behavior represents bistability resulting from the existence of two stable steady states separated by an unsteady state. In Figure 5 (A) the lower and upper branches of S-shaped curves correspond to the stable steady-states with low and high activity levels, respectively (i.e. low and high pAKT). For some parameter values desired bistability is lost as shown in Figure 5 (C).

In this paper we first extend the AKT model to include the cross-talk with the angiotensin signalling pathway. Next a similar parametric sensitivity analysis is performed to describe the effect of the cross-talk interaction parameters on both the steady-state and dynamic response characteristics of the system.

### Development of the New Dynamic Model

Since we focus on the relationship between diabetes and hypertension in this study, we have extended Wang's AKT model by including the interactions with the angiotensin signalling pathway. Using this new model, bistability and parameter sensitivity analysis is carried out to shed light on the key interactions and feedback loops that govern the development of diabetes and hypertension.

In order to facilitate the dynamic analysis, first we reduce the five-state dynamic model (1) – (5) to a two-state reduced-order model (see File S1 for the derivation):
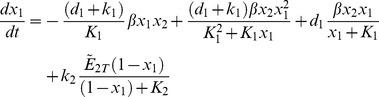
(10)

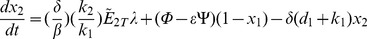
(11)where the normalized states are:
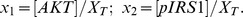



and the parameters are:



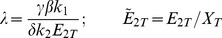


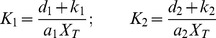



The above model is next modified by introducing the additional mass balances and interactions for the species *NO, ANG II, ROS* and *ONOO* which are involved in the cross-talk between AKT and angiotensin pathways (see Fig. 2). The new model is given by (see File S2 for derivation):
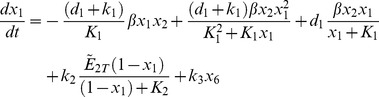
(12)

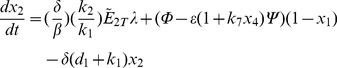
(13)

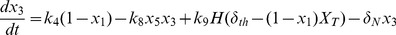
(14)

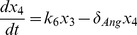
(15)

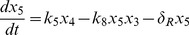
(16)

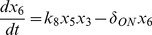
(17)where:
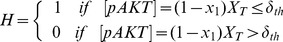
(18)



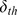
 is a threshold value for [*pAKT*].

and the states are:


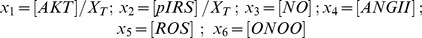


New model parameters 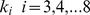 represent the strengths of the interactions between different states or nodes involved in the cross-talk and are labeled on the corresponding edges in Figure 4.

When bistability is lost and the level of *pAKT* stays persistently low, the glucose cannot be taken into the cell and high levels of glucose remain in blood. High glucose levels lead to GPR91 mediated activation of *NO* [41]. This hyperglycemia effect is expressed by the third term 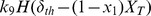
in the mass balance of *NO* given by equation (14). 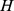
is a step function defined by (18). When 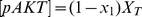 is below the threshold value of 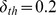 and hyperglycemia occurs, 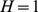; otherwise, 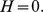 In equation (14) parameter 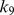 represents the rate constant for the production of *NO* during hyperglycemia. Parameters 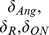
and 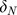 are the decay constants of *ANG II, ROS*, *ONOO* and *NO*, respectively.

The above cross-talk model is reproduced using the modeling tool CellDesigner and extracted in SBML (Systems Biology Markup Language) format (http://sbml.org/). SBML file is provided as a file S3.

## Results and Discussion

### Analysis of the AKT Model Without the Cross-talk

In order to establish a basis of comparison, we first present the dynamic performance of the AKT system without any of its interactions with the angiotensin signalling pathway. In this case, model equations reduce to (10) and (11). In [45] the same AKT system is studied but only steady-state response curves are provided. Here, in addition to the steady-state behavior, we present dynamic simulation results as well. For this purpose dynamic differential equations (10) and (11) are simulated with the following values of the model parameters:
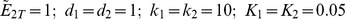


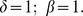



Biological systems possess a certain degree of robustness meaning that some desired properties hold against uncertainty such as variations in the kinetic parameters [51]. Due to lack of reliable data, true values for most of the above parameters are not available. However, bistability is known to be a robust property of the system that is preserved for a wide range of parameter values. The above chosen values represent one such set although they may not be the real values. Therefore chosen parameter values are biologically relevant as they characterize qualitatively the typical normal response behavior observed in real systems. In Figure 5 (A) we see that when the critical parameter 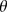 is chosen equal to 1, bistability exists. The dynamic response of the model for this normal phenotype is shown in Figure 5 (B). The dynamic trajectory shows how the system is able to switch between:

Steady State A:
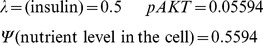
and

Steady State B:
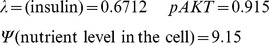



At State A, the cell has low nutrient level and requires glucose uptake. By stimulating insulin, the system switches to State B, where pAKT is activated and glucose is taken into the cell. Withdrawing insulin enables the switch back to low pAKT levels. Due to high sensitivity established by the positive and negative feedback actions, the required range of insulin change is quite narrow.

When 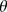 falls to negative values, e.g. 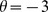, the system remains in the low pAKT state even if large quantities of insulin are supplied as shown in Figure 5 (C). Dynamic model's behavior confirms the steady-state predictions reported in [45] which indicates the loss of bistability for 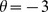. This resulting state is defined as the insulin resistant state where the glucose uptake is impaired due to persistently low pAKT activity. Disturbances that strengthen the negative feedback can cause the diabetes progression. For example mTOR is known to phosphorylate S6K which inhibits the function of IRS1 (see Figure 1). This is represented in the model by the negative feedback strength 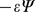. If mTOR activity becomes unregulated (e.g. due to prolonged exposure to insulin and genetic loss of tuberin TSC2 which down regulates mTOR), negative feedback dominates the positive feedback and 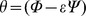
becomes negative and insulin resistance emerges. High blood glucose levels can eventually develop into type II diabetes. Also, due to less activated AKT, cells become more susceptible to apostosis.

Coupling of positive and negative feedbacks plays a significant role in the physiology of the system. Positive and negative feedback strengths should be balanced for a normal healthy operation. When positive feedback strength is increased to a higher level than its normal value or when the negative feedback is decreased, 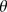 increases and the response curve becomes a one way switch, e.g. curve 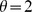 in Figure 5 (A). The system can switch from low pAKT to high pAKT level; however, once it reaches the high pAKT state, it stays there even if all insulin is removed. Persistently high pAKT levels drive uncontrolled cell proliferation, defined as cancer state.

**Figure 6:**
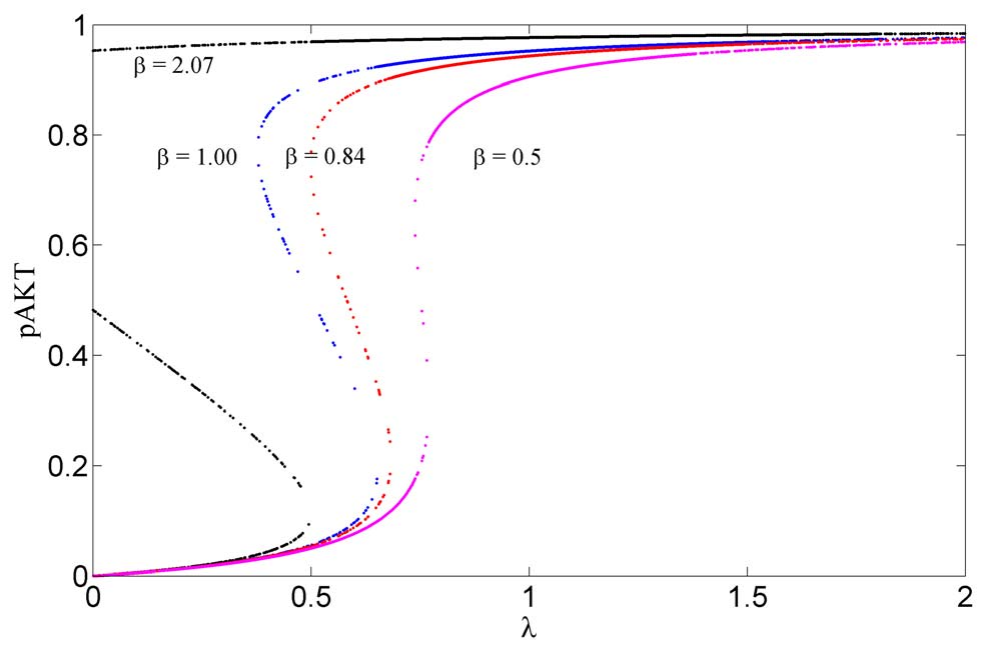
Steady State Response Curves for Different 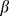 values. Bistability exists for values of 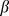 between 0.84 and 2.07. Larger values of 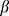 would correspond to the decreased function of PTEN. In this case pAKT stays at a high level once it is activated. This leads to elevated mTOR signalling and tumor growth.

The lipid protein phosphatase PTEN negatively regulates AKT activation and acts as tumor suppressor [52]. Mutations in PTEN gene result in various cancers. In our model the feedback strength 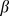 can be used to simulate the decrease/loss of PTEN function. Figure 6 shows the bifurcation diagrams for 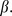 The system exhibits bistability for values of 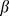 between 0.84 and 2.07. Large values of 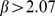 would correspond to the decreased function of PTEN. In this case pAKT stays at a high level once it is activated. This results in elevated mTOR signalling which can lead to preferential growth of tumor cells.

mTOR plays an important role in cell growth and proliferation. mTOR is a known target for the anticancer drug ramapycin [47], [48]. Inhibition of mTOR increases the pAKT production since 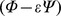 increases i.e. negative feedback effect of mTOR decreases. This reverses the insulin resistance. This is consistent with the literature, where it is stated that ramapycin reverses the insulin resistance [53]. mTOR inhibition by drugs lessens the effects leading to cancer through other pathways. However, as far as the insulin signalling pathway is concerned, mTOR inhibition weakens the mTOR mediated negative feedback to pIRS1, and account for persistent pAKT activation, driving uncontrolled cell proliferation. This can help explain why anticancer drugs that target mTOR inhibition are less successful than expected [49].

### Analysis of the AKT-NO Model

After analyzing the basic AKT system presented above, we next analyze the contributions of different regulatory feedback structures to the cross-talk between the AKT and Ang II signalling pathways. For this purpose, we simulate the dynamic models we have developed earlier. We are not able to compare our simulation results with other studies since there is no similar mathematical model for the cross-talk in the literature. However, we do provide a discussion of our model predictions in light of experimental observations reported in the literature. It is shown that the model is able to reproduce the known physiological behaviors.

The first cross-talk model includes the additional interaction of pAKT with NO. Thus, only

pAKT→NO→ONOO→pAKT negative feedback loop is added to the base AKT model. Interaction parameters 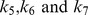
are set equal to zero. The resulting model consists of equations (12), (13), (14) and (17). Reactive oxygen species (ROS) enters the system as an independent stimulus (see Figure 4). Through the pathway pAKT→NO→ONOO→pAKT, pAKT enables NO production; NO and ROS react to form ONOO and ONOO in turn directly inhibits pAKT.

Five new parameters need to be specified in this case: 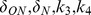
and 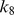. The following values were assigned to these parameters:



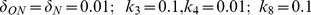
.

**Figure 7:**
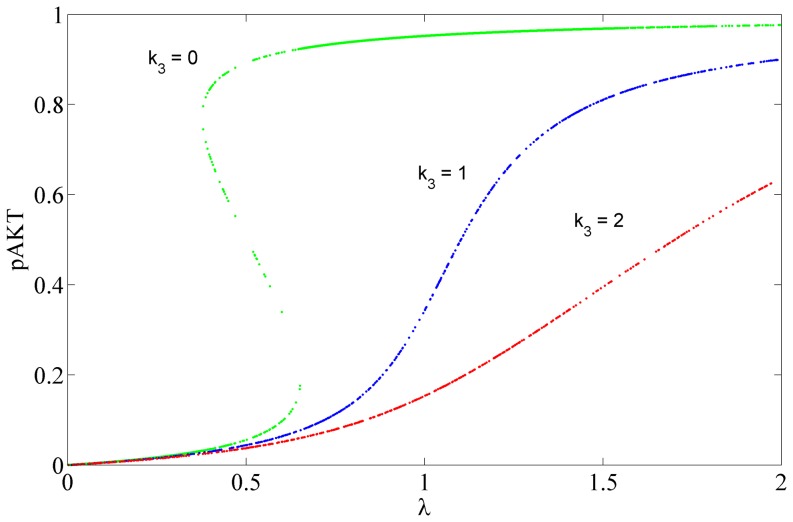
Steady State Response Curves for Different Levels of Inhibition by ONOO. The strength of inhibition is represented by the parameter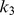. Increasing 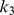 or inhibition reduces the sensitivity to insulin.

These nominal values were chosen because they preserve the bistability of the AKT signalling pathway and qualitatively produce the normal insulin cycle behavior. Parameter 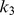
represents the strength of inhibition of pAKT by ONOO as expressed by the last term in equation (12). Next this parameter was perturbed from its nominal value to analyze the effect of ONOO inhibition on the insulin pathway. In Figure 7 steady-state response curves for pAKT are plotted for different values of 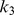. It is shown that as 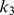
increases, bistability disappears and sensitivity to insulin decreases. This is in line with the fact that ONOO prevents AKT phosphorylation and inhibits its catalytic activity which promotes insulin resistance [37]. pAKT is positively influenced by insulin and PI3K as shown in Figure 4 by the edge that has strength β. At the same time, it is negatively influenced by ONOO as shown by the edge that has strength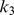. Thus, insulin sensitivity is adversely affected if inhibition by ONOO dominates the upregulation of pAKT by PI3K.

**Figure 8:**
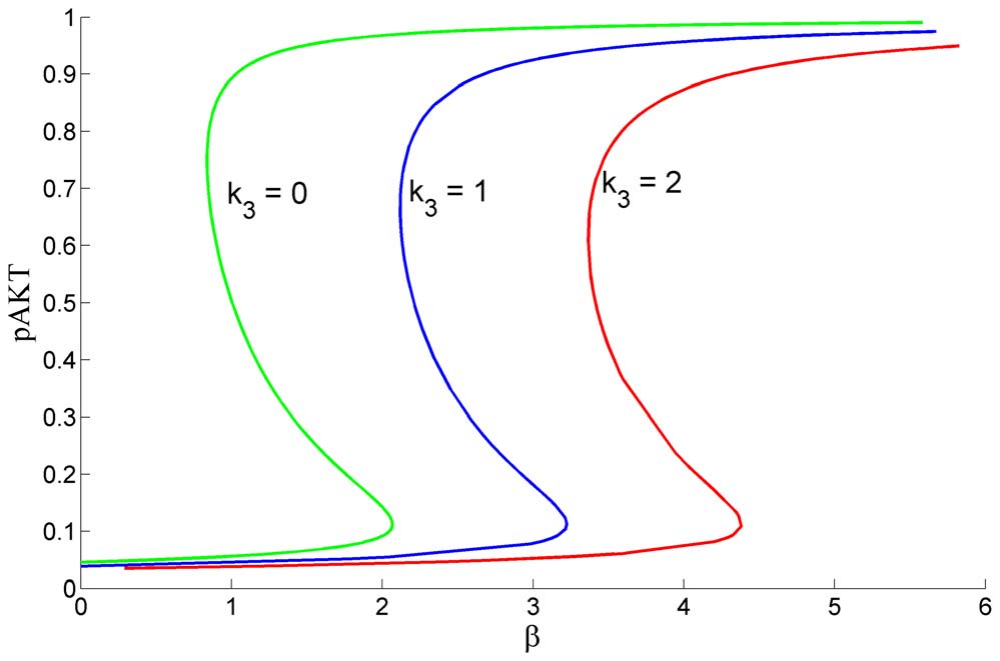
Bifurcation Diagram Showing Bistability Limits for β for Different k_3_ Values when λ = 0.5. As k_3_ increases, bistability is lost unless β increases to compensate the inhibitory action of ONOO by activating pAKT more.

Angeli et al [54] has presented a simple graphical method to detect bistability for biological positive feedback systems. Here we have applied this method to identify the range of values for
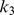
and β that guarantee bistability. The bistability limits for different 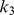
values are displayed in Figure 8. As k_3_ increases, bistability is lost unless β increases to compensate the inhibitory action of ONOO by activating pAKT more. For example, when 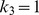, for β<2.07 there exists only 1 solution at a low pAKT level and the system is not bistable. However, for a slightly higher value 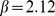, the system will recover the desired bistability. That is, changing the strength of the negative feedback to pAKT changes the limits of β that produce bistability. This suggests that for systems where there is a disturbance that enhances insulin mediated activation of pAKT, inhibition of pAKT by the negative ONOO feedback loop would recover the system back to bistability and prevent persistent activated pAKT state (cancer). Conversely, if β values are normal, increase of this negative feedback causes the system to lose its bistability characteristics. The system may reside in a low pAKT state and thus develop insulin resistance.

### Analysis of the ANGII-AKT Model

AngII-AKT Model is the full model that includes all the interactions between insulin and Ang II signalling pathways. Therefore interaction parameters 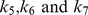 are no longer set to zero, and the model consists of all the equations (12) – (17).

The base scenario represents the normal phenotype where the bistability of the insulin signalling pathway is preserved in the presence of the cross-talk with the ANGII signalling system. In addition to the AKT system's parameter values chosen earlier, we have assigned the following values to the newly introduced parameters:
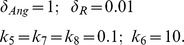



These values can be considered biologically relevant since the normal insulin cycle of the best case is maintained in the presence of the cross-talk as shown next.

**Figure 9:**
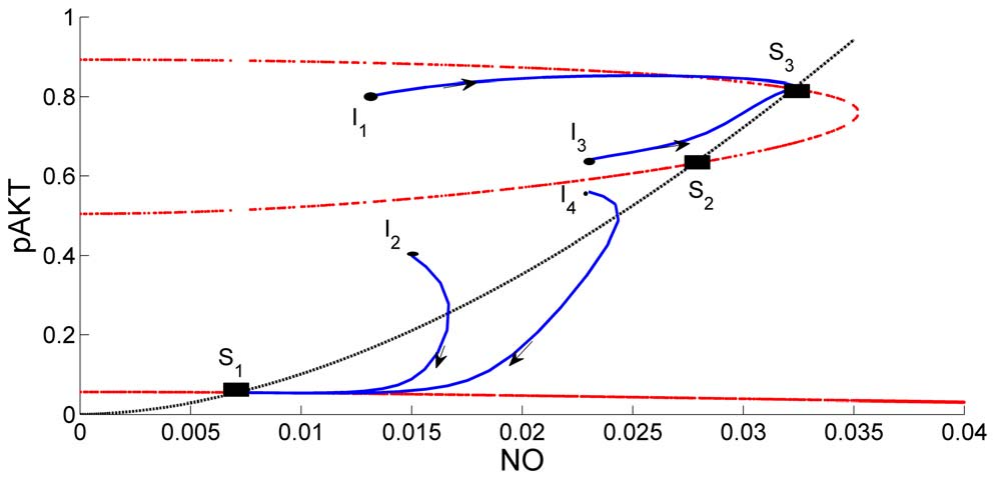
Steady State Solutions and Trajectories Predicted by ANGII-AKT Model. S_1_, S_2_ and S_3_ denote the three steady-states when insulin level λ = 0.5. The steady-states are at the intersection of red curves and black curve. Solid blue lines are the trajectories that start from different initial conditions I_1_, I_2_, I_3_ and I_4_. NO and pAKT both exhibit the desired bistability and switching dynamics behavior.

**Figure 10:**
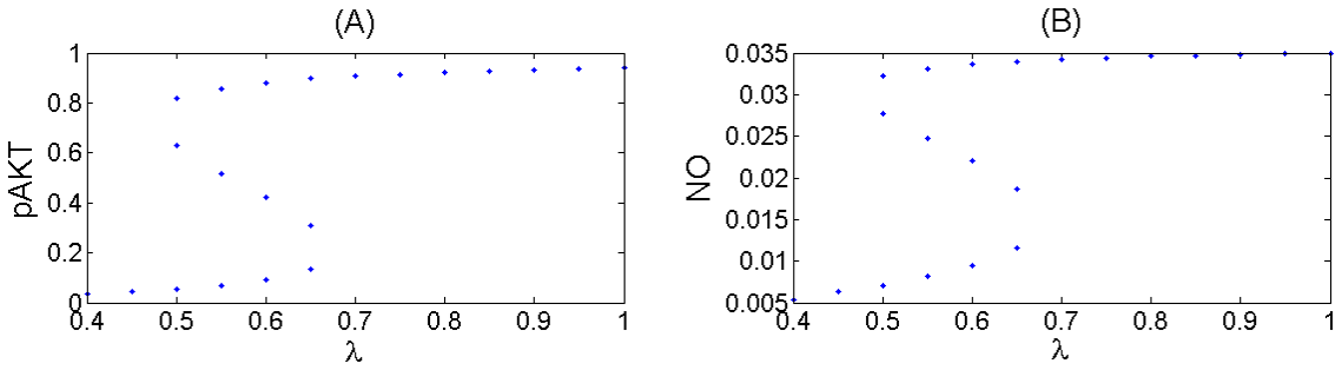
State Solution Curves for the Base Scenario Showing Joint Bistability for Various Insulin Levels. (A) for pAKT and (B) for NO.

In Figure 9 the intersections of the red curves and black curve give the steady state solutions for the selected parameter values. At these points the mass balance for NO (black curve) and mass balance for pAKT (red curve) are jointly satisfied. The curves intersect at three steady states: S_1_ (low pAKT), S_2_ and S_3_ (high pAKT). Dynamic simulations are carried out starting from initial points, I_1_, I_2_, I_3_, and I_4_. Steady State S_3_ is reached when the dynamic simulation starts from nearby initial conditions I_1_ and I_3_. Steady State S_1_ is reached when the dynamic simulation starts from nearby initial conditions I_2_ and I_4_. Steady states S_1_ and S_3_ are stable, whereas S_2_ is unstable. Steady-state solutions for different insulin levels λ are plotted in Figure 10. In addition to the known bistability characteristic of pAKT in the insulin pathway[45], our model predicts a similar behavior for NO since NO production depends on activation of pAKT. In conclusion both NO and pAKT are found to exhibit the desired bistability and switching dynamics behavior.

### Ang II inhibits the insulin metabolic signalling

**Figure 11:**
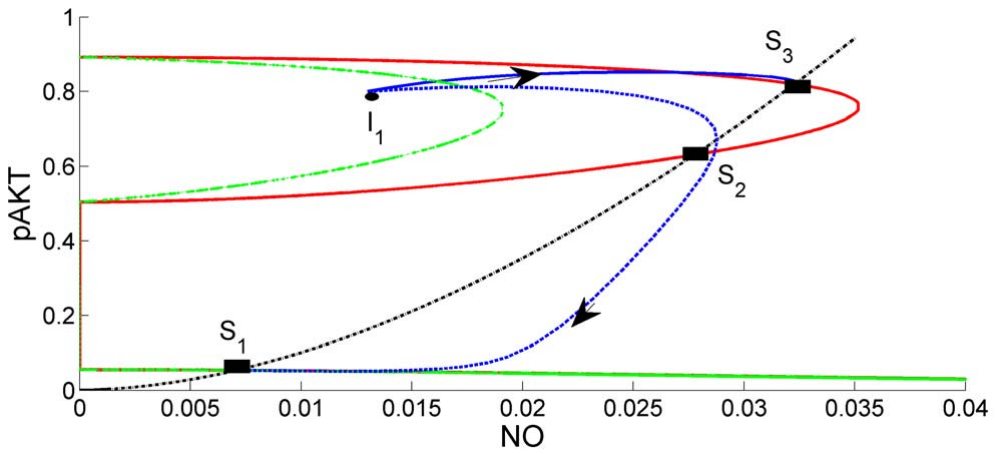
Comparison of Base Case (k_7_ = 0.1) with the Case k_7_ = 100. Red curves are the steady-state curves for the base case (k_7_ = 0.1). Green curves are the same curves when k_7_ = 100. Intersections of red and green curves with the black define the steady-states for the two cases. Solid blue curve is the state trajectory for the base case. Dashed blue curve is the state trajectory for the case when k_7_ = 100. As the effect of Ang II is increased by increasing k_7_, the system loses bistability. Trajectories converge to the single steady-state S_1_ at low levels of pAKT and NO.

It is known that angiotensin II inhibits the insulin metabolic signalling by promoting S6K1-mediated IRS-1 serine phosphorylation [40]. In order to simulate this negative effect, feedback parameter k_7_ is increased from 0.1 to 100 in the model. Such a large perturbation is reasonable since abnormal behavior and diseases are expected to develop due to large perturbations. As the effect of Ang II is increased by increasing k_7_, pAKT decreases (original red curve in Figure 11 shifts to the left as green) and the system loses bistability. A single steady-state S_1_ emerges where both pAKT and NO are low. The trajectories starting from different initial conditions converge to this steady state. This is shown for one initial condition I_1_ in Figure 11. As a result of low levels of pAKT and NO at the single steady-state S_1_, both insulin sensitivity and vasodilation are impaired. Thus, the model correctly predicts angiotensin II inhibition of the insulin pathway and associated insulin resistance [40].

### Proper balance must be maintained between pAKT and NO

**Figure 12:**
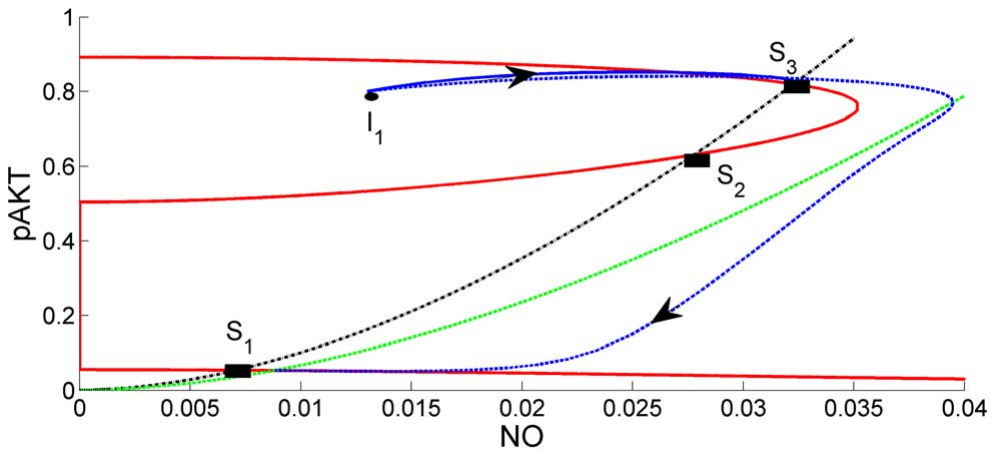
Comparison of Base Case (k_4_ = 0.01) and the Case k_4_ = 0.015. Red curves are the steady-state curves for both cases. Black curve is the steady-state curve for the base case (k_4_ = 0.01). Green curve is the new curve when k_4_ = 0.015. Intersections of red curves with the black define the steady-states for the two cases. Solid blue curve is the state trajectory for the base case. Dashed blue curve is the state trajectory for the case when k_4_ = 0.015. NO production by pAKT is enhanced by Increasing the value of parameter 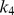 increases availability of NO and production of ONOO which impairs insulin signalling. Bistability is lost and the system settles to the single steady-state S_1_ where both pAKT and NO are low. In normal conditions (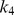 = 0.01), pIRS1-pAKT-pIRS1 and pAKT-NO-pAKT feedback loops work in coordination to maintain both insulin sensitivity and the right amount of NO by switching between S_1_ and S_3_ as necessary.

NO production by pAKT is enhanced by increasing the value of parameter 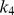 from its nominal value 0.01 to 0.015. Figure 12 shows the comparison with the base scenario. Original black curve shifts to the right (green) due to increased availability of NO. Elevated NO levels lead to activation of Ang II and production of ONOO which in turn impairs insulin signalling. In turn less NO is produced. As a result, bistability is lost and the system settles to the single steady-state S_1_ where both pAKT and NO are low. Our model suggests that in normal conditions (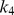 = 0.01), pIRS1-pAKT-pIRS1 and pAKT-NO-pAKT feedback loops work in coordination to maintain both insulin sensitivity and the right amount of NO by switching between S_1_ and S_3_ as necessary.

### Hyperglycemia overstimulates Angiotensin II

When bistability is lost and the level of pAKT stays persistently low, the glucose cannot be taken into the cell and high levels of glucose remain in blood. High glucose levels lead to GPR91 mediated activation of NO. The increase in NO can explain the presence of glomerular hyperfiltration observed in diabetes [42]. Hyperglycemia induced high NO levels stimulate ANG II [41] which in turn decreases NO by converting it to ONOO and imparing insulin signalling. At the same time production of Ang II increases blood pressure. Thus hyperglycemia contributes to hypertension development.

**Figure 13:**
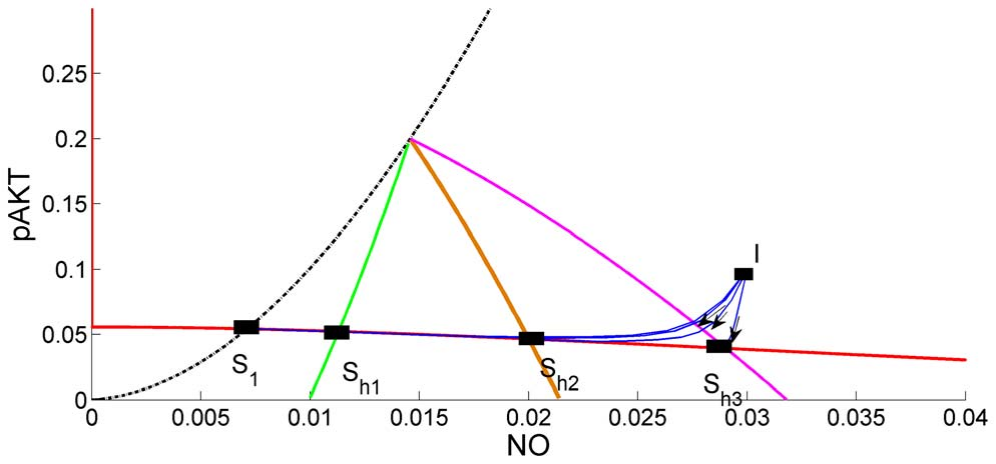
Effects of Hyperglycemia. Points S_1_, S_h1_, S_h2_, S_h3_ represent the steady-states. At these steady states NO levels increase as the parameter 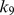
or hyperglycemia effect increases. Black, green, brown and red curves for k_9_ = 0, 0.005, 0.02 and 0.04, respectively. Intersections of these curves with the low-pAKT steady-state curve (red) define the steady-states for different levels of hyperglycemia. Dynamic simulations starting from initial condition I converge to different steady-states. Solid blue curves are the state trajectories for different k_9_ values. pAKT stays below the threshold value 0.2 and increasing 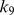 or hyperglycemia shifts the steady state to higher NO levels.

Hyperglycemia's effect can be simulated by choosing the model parameters so that bistability does not exist and by increasing the hyperglycemia parameter 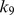. Figure 13 shows the hyperglycemia region where pAKT stays below the threshold value 0.2. At the same time it is seen that increasing 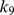 or hyperglycemia shifts the steady state to higher NO levels leading to increased production of Ang II

## Conclusions

In this work, we have proposed a new mathematical model that is able to predict known physiological behaviors of normal and diseased states (including diabetes, hypertension) that are governed by the interaction of insulin and angiotensin II signalling pathways. This model extends the AKT signalling model of Wang [45] by including its interactions with the angiotensin signalling pathway.

The model is developed by making use of significant amount of available biological knowledge. Using this new model, bistability and parameter sensitivity analysis is carried out to reveal the interactions and feedback loops, and their parameter regions, which are significant in disease development such as cancer, diabetes, and hypertension. Simulations show that the model is able to predict the observations that many researchers have reported in the past. As such, it provides analytical insight to improve our understanding of an important biological system.

The normal regulatory response of insulin signalling is bistable so that AKT can switch between low and high levels. It is shown that the interaction parameters in the model, the balance of positive and negative feedback strengths are important in maintaining this bistability. pAKT regulates pIRS1 both positively and negatively. For a normal bistable response, the effect of positive feedback should be greater than the negative feedback. When the difference between the feedback strengths is negative, the system loses bistability and a large amount of insulin needs to be supplied to activate pAKT. In severe insulin resistant cases the system settles in low pAKT state and glucose uptake is impaired, thus characterized as diabetes. Ang II can impair insulin signalling through several mechanisms: by direct inhibition of pAKT by ONOO, by directly inhibiting pIRS1 or by increasing the negative feedback to pIRS1 via activating mTOR. Ang II is involved in both physiological and pathological blood pressure and cell proliferation respectively. Systems with overly active Ang II as a result of a disfunction (e.g diseased kidneys can stimulate Ang II) can induce diabetes through these mechanisms.

Insulin also mediates vasodilation by stimulating NO production. NO in turn activates Ang II and Ang II inhibits pIRS1 as explained earlier. The events following stimulation of NO by pIRS1, activation of Ang II by NO and inhibition of pIRS1 by Ang II forms a negative feedback loop. We also found that NO has also bistable characteristics and shows switching dynamics together with pAKT.

On the other hand, when pAKT is overly active (e.g. through enhanced activation of pAKT by pIRS1), uncontrolled cell proliferation appears and the persistent high pAKT level is characterized as cancer. In a cancer state, therapeutic actions to decrease pAKT can be taken.

Activation of pAKT is also significant for vasodilator actions of insulin as pAKT stimulates NO production. When insulin signalling is impaired, vasodilation is impaired as well. To maintain blood pressure within narrow limits NO and Ang II should be balanced. NO activates Ang II and Ang II leads to consumption of NO to produce ONOO. ONOO and Ang II in turn impair insulin signalling and lead to further decrease in NO. The effects should be balanced to keep NO and Ang II at desired levels to maintain blood pressure.

Ang II also activates pERK [37]and Ang II activated pERK inhibits pIRS by serine phosphorylation. We have modelled these additional effects as well [55] but those results are not reported here for two reasons. First, the main interactions of the Ang II-AKT pathway targeted in this paper are not affected by not considering these interactions. Second the model is kept simple in order not to complicate the analysis and obscure the physical insights.

Investigating the regulatory and diseased mechanism at system level provides a valuable understanding of complex interacting pathways. A fine balance of interactions between the signalling agents should be maintained to avoid diseases.

Finally, due to lack of reliable data on the model parameters, future experimental measurements to estimate the key interaction parameters identified in this study will be invaluable to validate the proposed model firmly and improve our understanding of the biological network dynamics.

## Supporting Information

File S1 **Derivation of the two-state reduced order dynamic AKT model.**
(DOCX)

File S2 **Derivation of the six-state ANGII-NO model.**
(DOCX)

File S3 **SBML file for the model.**
(XML)
