# Supplementary material for: Regulatory Networks and Complex Interactions between the Insulin and Angiotensin II Signalling Systems: Models and Implications for Hypertension and Diabetes
Source: PLoS One. 2013 Dec 31;8(12):e83640. doi: 10.1371/journal.pone.0083640 (PMC3882141; doi:10.1371/journal.pone.0083640)
Supplement: File S1 — Derivation of the two-state reduced order dynamic AKT model. (DOCX) [file pone.0083640.s001.docx]

**S1: Derivation of the two-state reduced order dynamic AKT model.**

Define the following variables:

$$x_{1}=\left[AKT\right]/X_{T}; x_{2}=\left[AKT:E_{1}\right]/X_{T}; x_{3}=\left[pAKT\right]/X_{T}; x_{4}=\left[pAKT:E_{2}\right]/X_{T}; x_{5}=\left[pIRS\right]/X_{T}$$

where $X_{T}=\left[AKT\right]+\left[pAKT\right]$

Then Equations (1) - (5) can be expressed in terms of the above variables:

$\frac{dx_{1}}{dt}=\frac{-(d_{1}+k_{1})}{K_{1}}x_{1}(\beta x_{5}-x_{2})+d_{1}x_{2}+k_{2}x_{4}$ A1.1

$\frac{dx_{2}}{dt}=\frac{(d_{1}+k_{1})}{K_{1}}x_{1}(\beta x_{5}-x_{2})-(d_{1}+k_{1})x_{2}$ A1.2

$\frac{dx_{3}}{dt}=\frac{-(d_{2}+k_{2})}{K_{2}}x_{3}(\tilde{E}_{2T}-x_{4})+d_{2}x_{4}+k_{1}x_{2}$ A1.3

$\frac{dx_{4}}{dt}=\frac{(d_{2}+k_{2})}{K_{2}}x_{3}(\tilde{E}_{2T}-x_{4})-(d_{2}+k_{2})x_{4}$ A1.4

$\frac{dx_{5}}{dt}=(\frac{\delta}{\beta})(\frac{k_{2}}{k_{1}})\tilde{E}_{2T}\lambda+(\Phi-\varepsilon\Psi)x_{3}-\delta(d_{1}+k_{1})x_{5}$ A1.5

where $\tilde{E}_{2T}=E_{2T}/X_{T}$ and $\lambda= \frac{\gamma\beta k_{1}}{\delta k_{2}E_{2T}}$.

Assuming that pseudo steady-state holds for the AKT and pAKT complexes, A1.2 and A1.4 are set equal to zero and solved for:

$x_{2}=\frac{\beta x_{5}x_{1}}{K_{1}+x_{1}}$ and A1.6

Since $X_{T}=\left[AKT\right]+\left[pAKT\right]$, it also follows that $x_{1}+x_{3}=1$ or

$x_{3}=1-x_{1}$ A1.7

Substituting A1.6 and A1.7 into A1.1-A1.5 one obtains:

A1.8

$\frac{dx_{5}}{dt}=(\frac{\delta}{\beta})(\frac{k_{2}}{k_{1}})\tilde{E}_{2T}\lambda+(\Phi-\varepsilon\Psi)(1-x_{1})-\delta(d_{1}+k_{1})x_{5}$ A1.9

Renaming $x_{5}$ as $x_{2}$one gets Equations (10) and (11).
