# Supplementary material for: Regulatory Networks and Complex Interactions between the Insulin and Angiotensin II Signalling Systems: Models and Implications for Hypertension and Diabetes
Source: PLoS One. 2013 Dec 31;8(12):e83640. doi: 10.1371/journal.pone.0083640 (PMC3882141; doi:10.1371/journal.pone.0083640)
Supplement: File S2 — Derivation of the six-state ANGII-NO model. (DOCX) [file pone.0083640.s002.docx]

**S2: Derivation of the six-state ANGII-NO model.**

The chemical reactions follow from the interaction of species shown in Fig.4. Corresponding mass action expressions including the reaction rates are given in this Supplement S2. In transient mass balances for individual species second-order reaction terms appear when there are two species involved in the reaction as in the case of conversion of *NO* to *ONOO* by the action of *ROS* which is expressed by$k_{8}\left[ROS\right]\left[NO\right]$. Therefore the differential equations for the species *ONOO, NO* and *ROS* contain second-order nonlinear terms. Step function *H* is introduced to model the hyperglycemia effect.  When *pAKT* is below a threshold value, hyperglycemia occurs and this leads to high *NO* levels. Thus *H* function appears only in the differential equation for *NO*.

Mass action for $\left[NO\right]$gives:

$\frac{d\left[NO\right]}{dt}=k_{4}\left[pAKT\right]-k_{8}\left[ROS\right]\left[NO\right]+k_{9}H(\delta_{th}-\left[pAKT\right])-\delta_{N}\left[NO\right]$ A2.1

$H(\left[pAKT\right])=\left\{\begin{matrix} 1 if \left[pAKT\right]\leq\delta_{th} \\ 0 if \left[pAKT\right]>\delta_{th} \end{matrix}\right.$ A2.2

Parameters $k_{4},k_{8},k_{9}$ are rate constants ; $\delta_{N}$is the decay rate of *NO*. $\delta_{th}$ is a threshold value for $\left[pAKT\right]$. When $\left[pAKT\right]$ is below this low value, hyperglycemia effect becomes active.

Similarly, mass actions can be written for the other species (see the interaction of species in Fig.4.) :

$\frac{d\left[ONOO\right]}{dt}=k_{8}\left[ROS\right]\left[NO\right]-\delta_{ON}\left[ONOO\right]$ A2.3

$\frac{d\left[ROS\right]}{dt}=k_{5}\left[ANGII\right]-k_{8}\left[ROS\right]\left[NO\right]-\delta_{R}\left[ROS\right]$ A2.4

$\frac{d\left[ANGII\right]}{dt}=k_{6}\left[NO\right]-\delta_{Ang}\left[ANGII\right]$ A2.5

Parameters $\delta_{Ang},\delta_{R},\delta_{ON}$ are the decay constants of *ANG II, ROS*, *ONOO*, respectively.

By defining

$$\begin{matrix} x_{3}=\left[NO\right] ; x_{4}=\left[ANGII\right] ; x_{5}=\left[ROS\right] ; x_{6}=\left[ONOO\right] \\ \end{matrix}$$

Equations A2.1-A2.4 become equations (14)-(17).

Equation (12) follows from adding ONOO inhibition of pAKT as a new (last) term to equation (10):

$\frac{dx_{1}}{dt}= -\frac{\left(d_{1}+ k_{1}\right)}{K_{1}}\beta x_{1}x_{2}+\frac{\left(d_{1}+ k_{1}\right)\beta x_{2}x_{1}^{2}}{K_{1}^{2}+K_{1}x_{1}}+d_{1}\frac{\beta x_{2}x_{1}}{x_{1}+ K_{1}}+ k_{2}\frac{\tilde{E}_{2T}\left(1-x_{1}\right)}{\left(1-x_{1}\right)+ K_{2}}+ k_{3}x_{6}$ A2.6

where $k_{3}x_{6}$ stands for activation of $\left[AKT\right]$ (or inactivation of $\left[pAKT\right]$) by $x_{6}=\left[ONOO\right]$.

Finally equation (13) follows from adding activation of mTOR by ANGII to equation (11):

$\frac{dx_{2}}{dt}=(\frac{\delta}{\beta})(\frac{k_{2}}{k_{1}})\tilde{E}_{2T}\lambda+(\Phi-\varepsilon(1+k_{7}x_{4})\Psi)(1-x_{1})-\delta(d_{1}+k_{1})x_{2}$ A2.7

The added term is $-k_{7}\Psi\left[ANGII\right]\left[pAKT\right]=-k_{7}x_{4}\Psi(1-x_{1})$. This added negative feedback term is due to activation of mTOR by ANGII and is proportional to both pAKT level, the nutrient level $\psi$ and ANGII. As a mechanism we assumed that nutrients and ANG II promote the association of Rheb-GTP with mTOR [39]. In equation (11), mTOR activity and thus the negative feedback $-\psi\left[pAKT\right]$ is only proportional to pAKT and nutrient level [44]. Here mTOR activity also depends on ANGII.
